# Seismic Experimental Study on New-Type Composite Exterior Wallboard with Integrated Structural Function and Insulation

**DOI:** 10.3390/ma8063732

**Published:** 2015-06-19

**Authors:** Shaochun Ma, Nan Jiang

**Affiliations:** 1 School of Civil Engineering, Tianjin University, Tianjin 300072, China; E-Mail: mashaochun@tju.edu.cn; 2 Key Laboratory of Coastal Civil Engineering Structure and Safety, Tianjin University, Ministry of Education, Tianjin 300072, China

**Keywords:** gypsum-concrete composite, exterior wallboard, insulation, seismic experiment, hysteresis curve, stiffness

## Abstract

In order to evaluate the seismic performance of new-type composite exterior wallboard, a total of six exterior and interior wallboards were incorporated in the experiment of seismic performance. Seismic performance such as the stress process, damage mode, hysteresis and skeleton curve, load-carrying and ductility coefficient, damping and energy dissipation, stiffness degradation as well as material strain of the exterior wallboards were analyzed with emphasis and compared with interior wallboards. Results of the experiment and analysis showed that both interior and exterior wallboards exhibited outstanding seismic performance. Due to the existence of insulation layer and externally bonded single gypsum board, the capacity of elastoplastic deformation and seismic energy dissipation of the exterior wallboards was improved and each seismic performance indicator of the exterior wallboards outperformed the interior wallboards.

## 1. Introduction

Within the background of global warming, decline in the quality of living environments and significant reduction of energy resources, energy-saving and protection of environments have become hot topics in countries all around the world [1]. According to the statistics of energy consumption in each country, the energy consumed in building and construction takes a large proportion of the total energy consumption. Walls contribute significantly to the effect of insulation of the building and are the key to resolving the issue of energy-saving of buildings to scientifically improve the enclosing structure and heating facilities of the building. Comparing different kinds of wall insulation approaches (external insulation of external wall, internal insulation of external and external wall sandwich insulation *etc*.), the advantages of external insulation of external walls are prominent, and they have been promoted and applied in most countries as an approach towards energy saving of buildings. Generally, the exterior of the external insulating material is tiled or grouted with cement and sand. However, the insulation system adapting such an approach is unlikely to share similar service life as the building. Therefore, the development of a new type of wallboard and insulation system is imminent.

Gypsum is a type of building material that is green and environmental friendly [2], which is one of the three main binding materials together with cement and lime. Among the three materials, gypsum is widely adapted in buildings due to its superior and special performance. The energy consumption during calcination for gypsum is merely 1/4 of that for cement and 1/3 for lime. In a prescribed environment, gypsum is capable of absorbing and releasing moisture and adjusting room humidity. Gypsum is a type of porous material that is nontoxic and the pores are effective in providing an insulating effect. The thermal conductivity of gypsum is only 1/5 of that of concrete and thus it is beneficial for the energy-saving of buildings. Gypsum (calcium sulphate dehydrate, CaSO_4_•2H_2_O) has up to 21% crystalized water and 70% calcium sulfate whose melting temperature is over 1400 °C. As a fact, gypsum can be considered as an ideal fire resistant material which postpones the spread of fire. The addition of fiberglass and additives into gypsum ensures building panels with good performance. The modified gypsum board has improved load-carrying capacity and shear strength compared to normal gypsum boards. Rapid fiberglass gypsum wallboard and the related structural system was the earliest new-type advanced building product developed in Australia [3]. The rapid gypsum wallboard is a type of fiberglass gypsum hollow panel which can be manufactured in factories on a large scale. The standard wall thickness is 120 mm and the gypsum cavity has a dimension of 94 mm × 230 mm. Rapid wallboard is mainly used as non-load-bearing partition wall or enclosing structure [4]. It has the advantages of being lightweight, energy-saving, sound insulating and environmentally friendly, and is easy to manufacture with a fast construction process. The addition of concrete, slag, sound insulation or thermal insulation into the gypsum cavity improves the load-carrying capacity as well as sound and thermal insulation of the wallboard. The gypsum board acts as permanent stay-in-place mould which reduces construction cost and it also participates in load-bearing [5]. For a long period of time, significant studies were conducted on rapid gypsum wallboards by scholars in Australia, China (Tianjin University and Shandong Construction Engineering Group) and India [6,7,8,9,10,11,12,13]. Due to the fact that concrete core gypsum wallboard is combined by concrete dense columns and gypsum boards, the load-carrying capacity and horizontal force resistance are relatively low. Meanwhile, the treatment of connections between board and board as well as board and structural columns is inconvenient. North-America and Europe have done a lot of research studies on the gypsum board, but basically on the gypsum board as a non-structural element of partitions [14,15,16,17]. Its force performance is relatively not very high.

According to the above conditions, based on the current design, a new type of gypsum-concrete composite exterior wallboard (CEW) is proposed in this study; *i.e.*, it is a new-type composite exterior wallboard with integrated structural function and insulation. One horizontal gypsum cavity is setup at every certain height in the direction of wall height on the vertical gypsum cavity. Asbestos board (or polystyrene board) insulation is setup at the exterior of the wallboard and bonded externally with a single panel of gypsum board. The complete gypsum wallboard product is manufactured in the factory and the gypsum cavity is poured with concrete when the wallboard is transported to the site, thereby forming a new type of gypsum-concrete composite exterior wallboard with lattice structure of concrete dense columns and beams and good insulation. The insulation system has distinct advantages of once-off formation with walls or structure as well as identical service life. In order to evaluate comprehensively the seismic performance of CEW, the new-type gypsum-concrete composite interior wallboard (CIW) with no insulation layer was used as comparative specimens. CEW and CIW specimens were designed according to trial engineering building in order to conduct seismic experimental studies. A series of studies were conducted with respect to the stress process, damage mode, load-carrying and deformation capacity, displacement ductility, energy dissipation, stiffness degradation as well as contribution of gypsum board and insulation panel. Results from this study are of great significance in advancing the evolution of new types of energy-saving and environmental friendly walls.

## 2. Experimental Section

### 2.1. Design of Specimens

**Figure 1:**
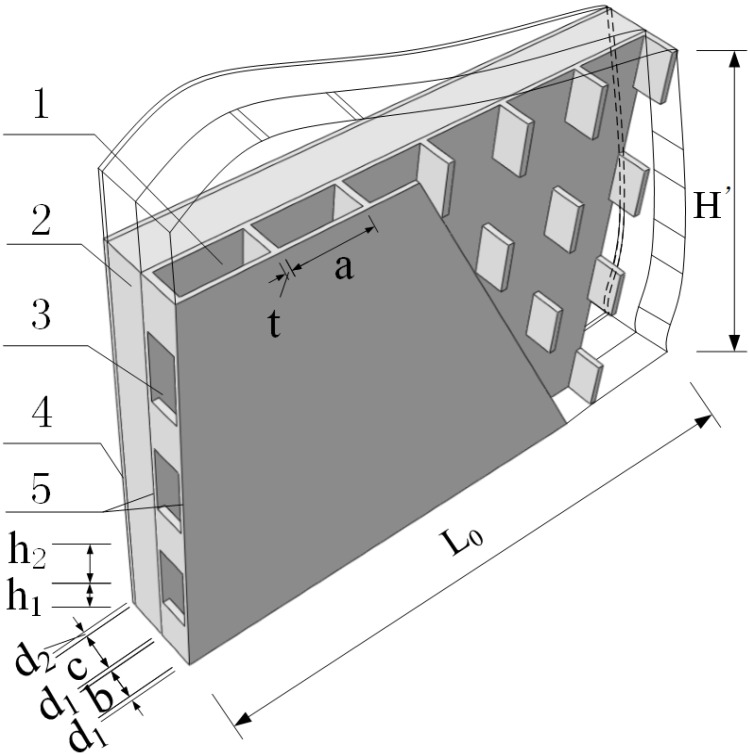
Schematic diagram of CEW multi-cavity gypsum board. 1: vertical gypsum cavity; 2: insulation board; 3: horizontal gypsum cavity; 4: externally bonded single gypsum board; 5: gypsum board.

The new type of gypsum-concrete composite wallboard system is mainly a combination of composite wallboard and connecting structural members. The gypsum boards used in this study were made from raw materials such as calcined gypsum, industrial by-product gypsum and non-alkali fiberglass. Fiberglass gypsum board products were made in the factory according to the specific mixture and standard sizes with addition of cement and chemical additives. Material test results showed that the compressive strength was 5.52 MPa and elastic modulus was 4350 MPa. The width of gypsum board for CIW was *b* + *d*_1_ = 120 mm. Gypsum thin boards with a thickness of *d*_1_ = 13 mm located at both sides and the center was a cavity surrounded by thin boards. Gypsum division boards with a thickness of *t* = 20 mm were setup every *a* = 230 mm in the direction of wall length and the height of the division boards was *h* = 160 mm. The gypsum division boards separated the cavity into vertical gypsum cavity with a dimension of *a* × *b* = 230 mm × 94 mm. A horizontal gypsum cavity with a dimension of *a* × *b* = 230 mm × 94 mm was setup every *h*_1_ long the direction of wall height. The gypsum boards were completed with the addition of polystyrene board with *c* = 120 mm as well as externally bonded single gypsum board with a thickness of *d_2_* = 13 mm, as shown in Figure 1. In the event of fire, the externally bonded single gypsum board and gypsum thin boards effectively stop the spreading of fire along the wall. The escaped steam acts as temporary fire extinguisher which eases the fire and prolongs the timeframe of heat transmission from the internal to external of the wall and thereby effectively protects the internal insulation and primary structure of the wall. We have made triaxial shear test of polystyrene board. Specimen is a diameter of 39.1 mm, high is 80 mm cylinder of polystyrene. *i.e.*, the results of material test of insulation board revealed that the compressive strength was 206 kPa and the elastic modulus was 2.3 MPa.

**Figure 2:**
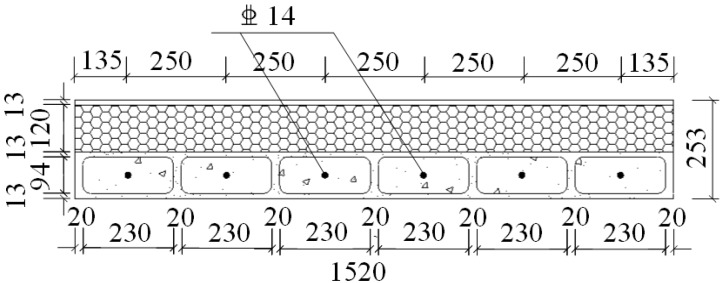
Plan view of CEW specimen.

**Figure 3:**
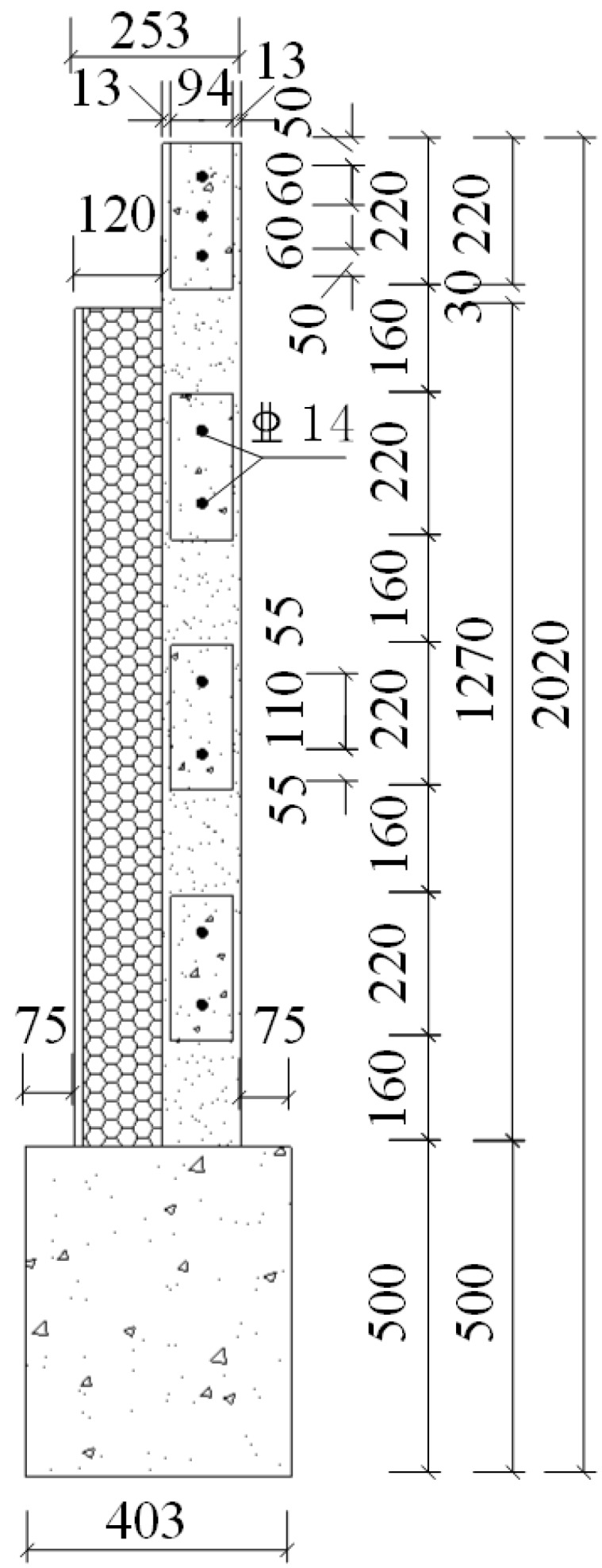
Side elevation of CEW specimen.

**Figure 4:**
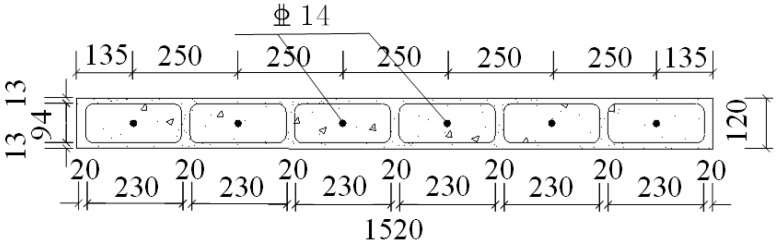
Plan view of CIW specimen.

**Figure 5:**
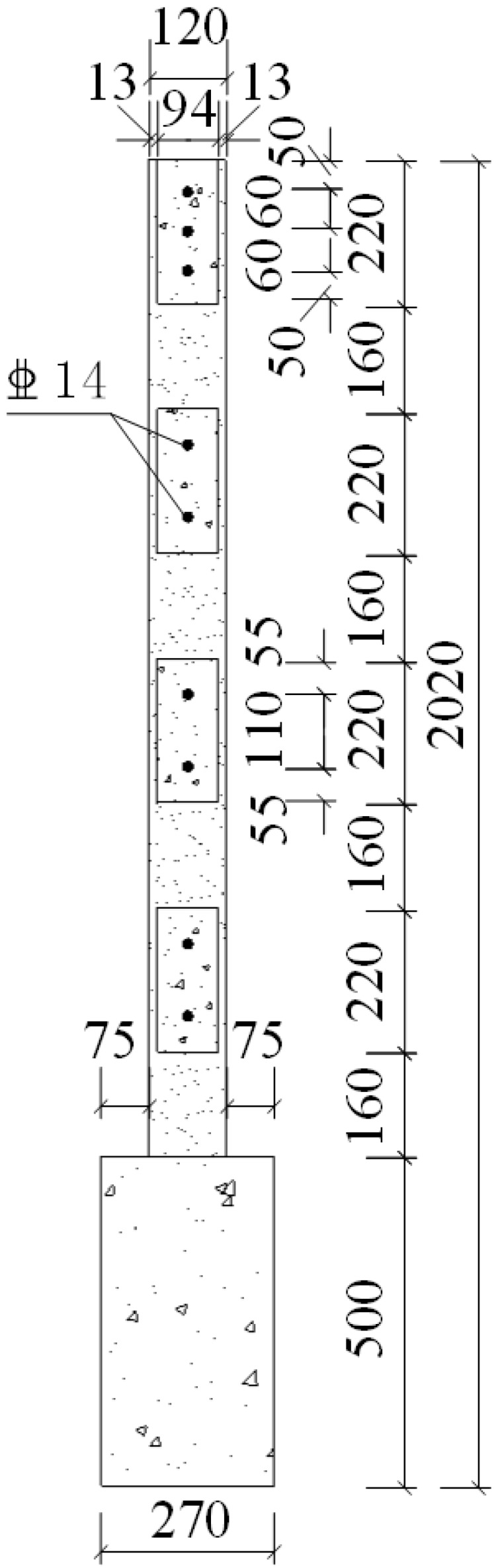
Side elevation of CIW specimen.

A total of six specimens were designed in this study. The notation for CEW specimens was respectively E-1, E-2 and E-3 while for CIW specimens, I-1, I-2 and I-3. Polystyrene board and externally bonded single gypsum board of CEW as the building envelope, the main consideration of its impact on the performance of the force wallboard structure. The inflection point position is in 1/2 layer height of laboratory building’s standard layer, and the bending moment is zero at the inflection point. So, taking the standard layer’s 1/2 storey as the height of the specimens. The specimens consist of wall, loading beam (120 mm × 220 mm) and ground girder (403(270) mm × 500 mm). C20 concrete and HRB400 reinforcement were used. Material test results showed that for concrete, *f_c_* = 24.63 MPa and *E_c_* = 2.74 × 10^4^ MPa; for steel reinforcement, *f_c_* = 654.00 MPa (D8), 669.45 MPa (D14) and 676.92 MPa (D20). 2D14 horizontal reinforcement was setup in each horizontal gypsum cavity and 1D14 vertical reinforcement was setup in each vertical gypsum cavity. The horizontal distributed reinforcement in the loading beam was 3D14. The longitudinal reinforcement of the ground girder was 4D20 and the stirrup was D8@200. The plan and side elevation views of CEW are demonstrated in Figure 2 and Figure 3. The plan and side elevation views of CIW are demonstrated in Figure 4 and Figure 5. Specimens are typically shear components which are almost square. The aspect ratio of the specimens had a profound influence on their failure mode. As the aspect ratio increased, bending or rocking like behavior and failure would occur, and the composite wallboard should be strengthened by increasing the edge reinforcement or structural column (*i.e.*, embedded column) at this time.

### 2.2. Layout of Strain Gauges

**Figure 6:**
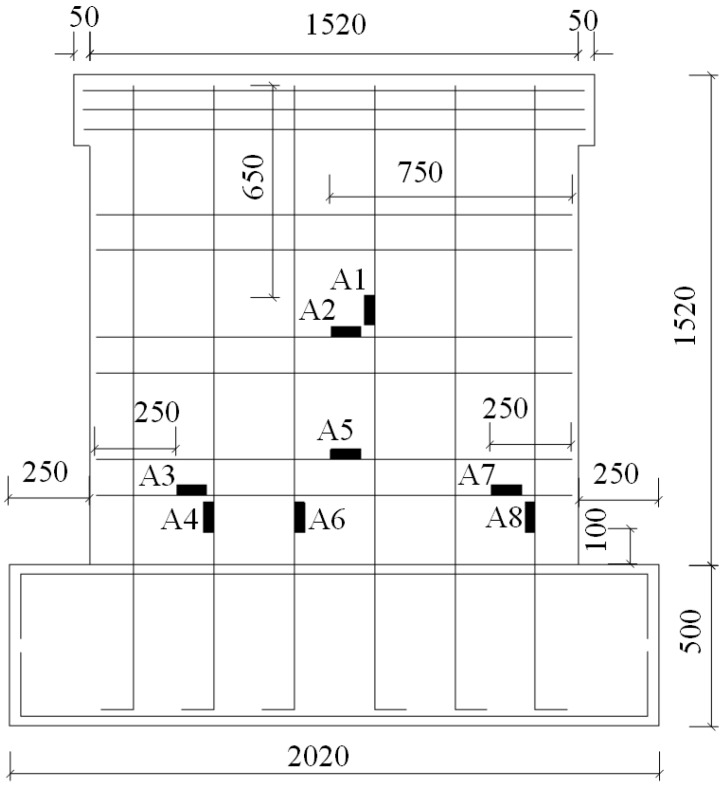
Layout of strain gauges on the reinforcement of CEW.

**Figure 7:**
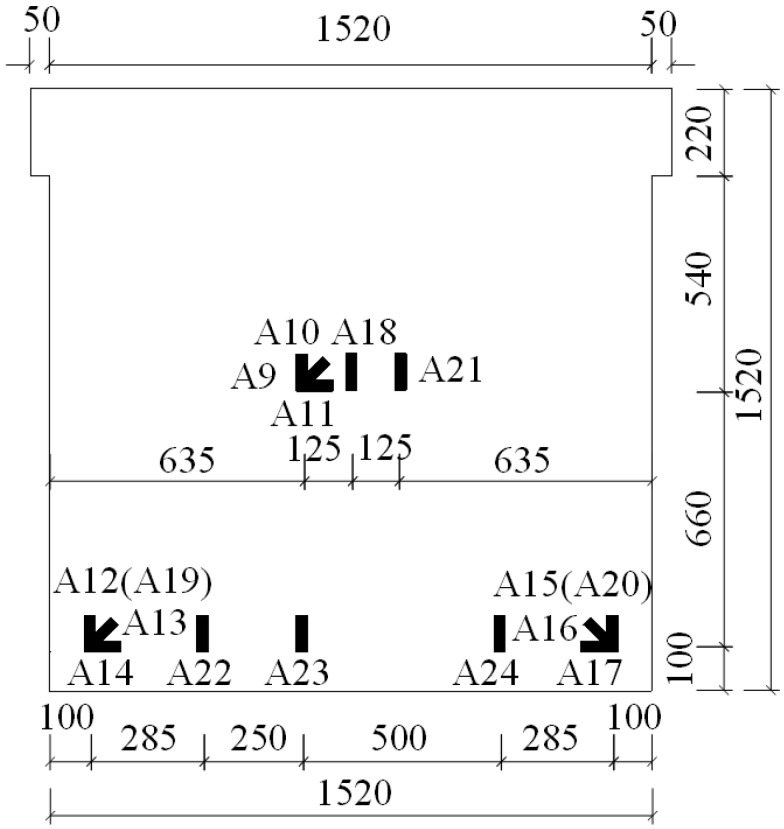
Layout of strain gauges on the concrete and gypsum boards of CEW.

During the experiment process, strain gauges were placed at key locations in order to obtain the material strain of CEW and CIW specimens. According to previous experience, the weak spots are located mainly in the middle or lower part of the wallboard. Therefore, respective strain gauges were setup on the middle and lower parts of steel reinforcement, concrete and gypsum boards, as shown in Figure 6 and Figure 7. Strain gauges A1 to A8 were placed on the reinforcement of CEW specimens, A9 to A20 were placed on gypsum boards (A18 to A20 were placed on the externally bonded single gypsum board), and A21 to A24 were placed on concrete. For CIW specimens, the strain gauges was denoted as B and the location as well as notation were identical to CEW specimens. For CIW specimens, due to the absence of insulation layer and externally bonded single gypsum board, strain B18 to B20 did not exist.

## 3. Experimental Instruments and Schedule of Loading

**Figure 8:**
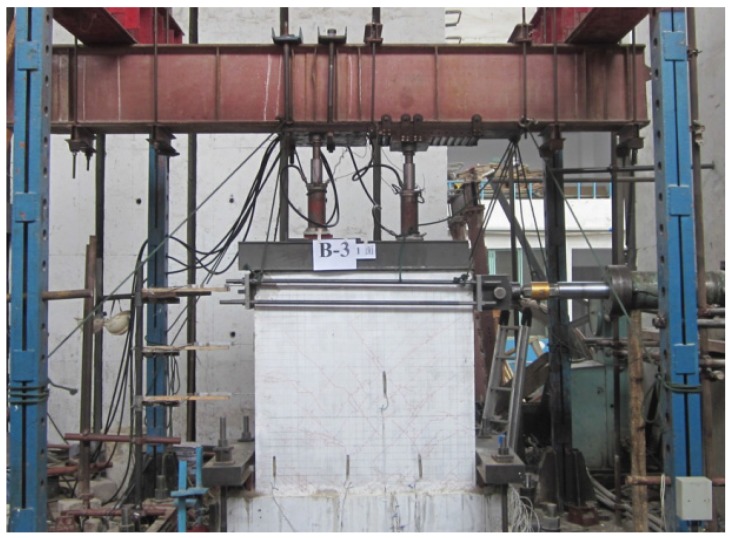
Onsite experimental loading instrument.

The experimental loading instruments are shown in Figure 8. According to Specification of Testing Methods for Earthquake Resistant Building (JGJ101-96) [18], quasi-static loading experimental approach was used to conduct seismic performance tests. Particularly, horizontal cyclic loading is the main approach of simulating seismic action. In this study, a 1000 kN horizontal push-pull lifting jack was used to apply horizontal cyclic load on the top of the specimen. Force and displacement control was employed for the vertical and horizontal loading, respectively. The schedule of loading was: load-controlled mode was used to conduct step cyclic loading and one cycle was loaded for each load step until the wallboard was yielded. After the wallboard was yielded, displacement-controlled step cyclic loading was used and three cycles were carried out for each displacement step. The load-distributing steel beam was used also to spread the vertical load (51.32 kN/m) of the two 500 kN hydraulic jacks on the upper surface of the specimens [19]. It was used to simulate the actual effective weight of the three floors. During the test, the vertical load was realized to a constant value in real time. Moreover, this load condition is aligned with that used to characterize the performance of walls [20,21,22]. Axial compression ratio (λ) is one of the main factors influencing the seismic capacity of the shear wall. It has good ductility and energy dissipation capacity when axial compression ratio is relatively small. “Code for seismic design of buildings” (GB50011—2010) limited the value of axial compression ratio (*i.e.*, λ_max_ = 0.50) [23] to ensure that the ductility of shear walls. The axial compression ratio of test composite wallboard is 0.075, which was less than the prescribed limit (λ_max_ = 0.50), so the results were ideal. If the vertical load was increased, the decrease ductility could lead to brittle failure of the specimens. During the entire process of the experiment, a data collection system was used to continuously collect the horizontal load, displacement and various material strain on the top of the specimens and describe the cracks produced on the specimens.

## 4. Experimental Results and Analysis

### 4.1. Stress Process and Mode of Damage

Due to the fact that the experimental process and mode of damage for CEW and CIW specimens were similar, Specimen E-1 was selected as a typical example in the discussion below.

**Figure 9:**
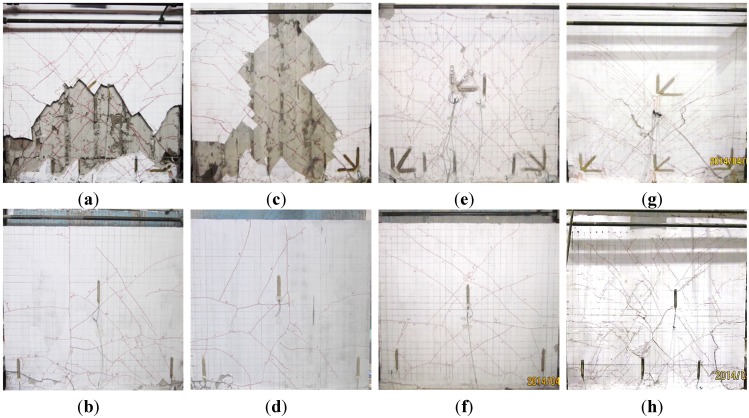
Failure of specimen. (**a**) E-1 (Front); (**b**) E-1(Back); (**c**) E-2 (Front); (**d**) E-2 (Back); (**e**) E-3(Front); (**f**) E-3 (Back); (**g**) I-2 (Front); (**h**) I-2 (Back).

(1) Preloading stage: during the 1st cycle, the specimen was loaded to 30.33 kN and 29.86 kN in the positive and negative direction, respectively. No obvious observation could be made and related instruments worked normally. The smooth process of preloading suggested that the experiment can be formally started [24,25].

(2) Initial cracking stage: during the 2nd cycle, the specimen was loaded to 50.11 kN and 50.35 kN in the positive and negative direction, respectively. Six micro-cracks in the direction of about 30° appeared at the bottom part at the front and the longest crack had a length of 370.00 mm. A horizontal micro-crack with a length of 300.00 mm appeared at the bottom part at the back of the specimen. It was suggested that during the initial cracking stage of the specimen, the cracks appeared firstly at the bottom part of the wall where the shear force was the largest.

(3) Cracking stage: during the 3rd cycle, the specimen was loaded to 100.51 kN and 102.96 kN in the positive and negative direction, respectively. Two diagonal 45° cracks appeared at the middle and bottom parts in the front and one bending and shear crack with a length of 180.00 mm appeared at the middle part which penetrated to the side of the specimen. During the 4th cycle, the specimen was loaded to 154.36 kN and 152.26 kN in the positive and negative direction, respectively. Four parallel 45° diagonal cracks with a length of 130.00 mm appeared at the middle part in the front and the cracks at the bottom part at the back widened with the appearance of new cracks. During the 5th cycle, the specimen was loaded to 200.49 kN and 200.79 kN in the positive and negative direction, respectively. Multiple 45° or 135° diagonal cracks which penetrated with each other appeared in the front and the primary diagonal cracks were basically formed. It was suggested that from the 3rd cycle to the 5th cycle, the number of cracks in the front and back increased and the cracks developed and connected with each other. Minor spalling of gypsum boards was witnessed.

(4) Yielding stage: during the 6th cycle, the specimen was loaded to 219.95 kN and 220.12 kN in the positive and negative direction, respectively. The general shape of primary diagonal cracks in the front were formed. At this moment, the specimen entered plastic stage and thus displacement-controlled loading was adapted. During the 7th cycle, the specimen was loaded to 4.11 mm and 4.05 mm in the positive and negative direction, respectively, and the width of cracks in the front and back continued to increase. During the 8th cycle, the specimen was loaded to 8.00 mm and 8.10 mm in the positive and negative direction, respectively. The 45° primary diagonal cracks in the front were widened and extended, and multiple primary diagonal cracks penetrating the entire wall surface were formed. During the 9th cycle, the specimen was loaded to 12.10 mm and 12.00 mm in the positive and negative direction, respectively. A part of the gypsum boards peeled off at the primary diagonal cracks. During the 10th cycle, the specimen was loaded to 16.10 mm and 16.20 mm in the positive and negative direction, respectively. A large part of the gypsum boards in the front were peeled off and thick 45° primary diagonal cracks were witnessed on the exposed concrete surface. It was suggested that from the 6th cycle to the 10th cycle, the specimen entered yielding stage and diagonal cracks in the front connected into honeycomb cracks. The amount of cracks did not increase dramatically but the length and width increased significantly. A large part of gypsum boards spalled at the joint of cracks. Both horizontal and vertical cracks were witnessed at the back.

(5) Damage stage: during the 11th cycle, the specimen was loaded to 21.40 mm and 20.10 mm in the positive and negative direction, respectively. The gypsum boards in the front peeled off severely, the concrete at the bottom right part was crushed and the reinforcement was bent. The cracks at the bottom part at the back were widened. During the 12th cycle, the specimen was loaded to 20.00 mm in the positive direction. The displacement increased dramatically then dropped quickly and the specimen failed. It was suggested that shear damage was the dominant damage mode for the specimen and the concrete at both sides was crushed. When the specimen failed, a large part of gypsum board in the front spalled (Figure 9a,c) or thick honeycomb cracks appeared (Figure 9e), together with peeling and hollowing. Obvious diagonal, horizontal and vertical cracks were witnessed at the back as shown in Figure 9b,d and f. The diagonal cracks were caused by shear force carried by the insulation board and externally bonded single gypsum board and the horizontal and vertical cracks were probably due to torsion as a result of eccentric force sustained by non-load-carrying insulation board and externally bonded single gypsum board.

The observation during cracking, yielding and damage stages for CIW specimens were similar with CEW specimens with only difference in values. Take Specimen I-2 as an example. The crack load was 60.11 kN, yield load was 206.40 kN and damage load was 249.43 kN. The failure mode was similar with the front face of CEW specimens. Only the pictures at failure for Specimen I-2 are given in this study, as shown in Figure 9g,h.

### 4.2. Hysteresis Loop of Horizontal Force-Top Displacement

**Figure 10:**
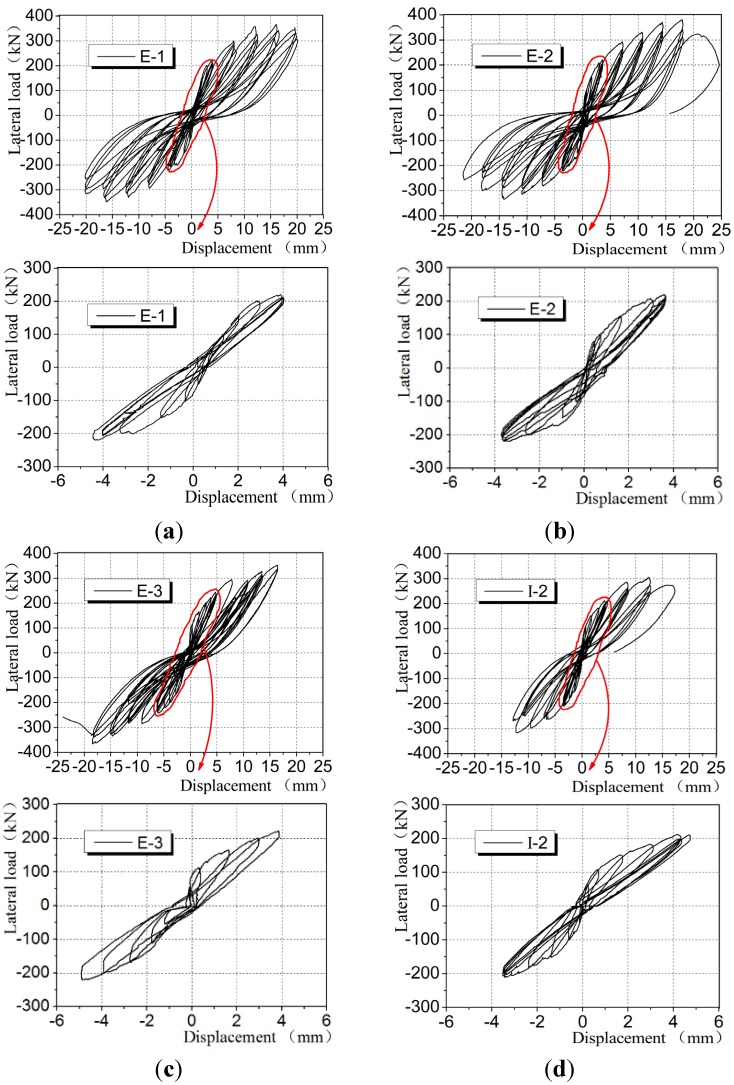
Hysteretic curves for CEW/CIW. (**a**) E-1; (**b**) E-2; (**c**) E-3; (**d**) I-2.

Figure 10 shows the hysteresis loop of horizontal force-top displacement for CEW and CIW specimens, in which typical CIW specimen I-2 was selected as comparative specimen.

It is evident that during the entire process of loading, the shape of hysteresis loops for both types of specimen experienced two stages but stable hysteretic characteristics were witnessed. For the first stage, during the initial loading period, the hysteresis loops for all CEW and CIW specimens were spindle-shaped (as shown in the local magnified diagram in Figure 10. At this moment, the effective area enclosed by the hysteresis loop was small and the variation of stiffness was small. For the second stage, with the increase of horizontal load, ongoing cumulative damage occurred in the specimen which led to stiffness degradation. The variation of shape of hysteresis loops was evident and they shifted to “S” shape. At this moment, the effective area enclosed by the hysteresis loop increased obviously. Residual deformation of specimens was witnessed and stiffness degradation was evident.

Comparative analysis of the hysteresis loops for CEW and CIW specimens showed that: for both types of wallboard, when unloaded to 0 during each load step, the hysteresis loops did not pass through the origin, which suggests that residual deformation occurred. Judging from the effective area surrounded by the hysteresis loops, those of CEW were larger than CIW specimens which indicates that the capacity of energy dissipation for CEW outperformed CIW. During the three cycles of the last few load steps, the reduction of maximum load corresponding to the hysteresis loops of CEW specimens was more significant than CIW, which suggests that the ductility of CEW was better than CIW.

### 4.3. Skeleton Curves

**Figure 11:**
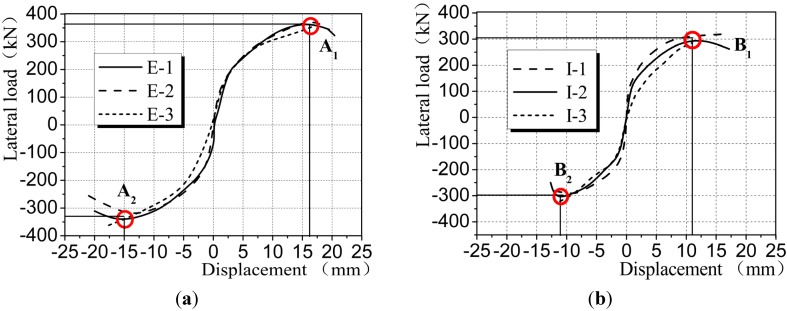
Skeleton curves of CEW/CIW. (**a**) CEW; (**b**) CIW.

The load-displacement skeleton curve was developed by connecting the peak values at each load step on the hysteresis loops [26]. It is evident from the comparison of skeleton curves in Figure 11 that during the initial loading stage, the skeleton curves for CEW and CIW increased straightly with a large slope but small displacement. The above findings show that CEW and CIW remained in an elastic stage. The wallboard entered an elastoplastic stage when the gypsum and concrete cracked. The skeleton curve bent to a certain degree, the displacement increased but the variation of stiffness was less significant. When the vertical reinforcement at both sides of the wallboard yielded, the degree of stiffness degradation for CEW and CIW specimens reduced with the increase of top load and displacement. Judging from the overall trend of skeleton curves, the increase of skeleton curves for CEW specimens was smoother and more even compared with CIW specimens, which suggests that the stress distribution of CEW was more even than CIW and the deformation was more stable. Judging from the ultimate load and displacement, CEW was much larger than CIW. From the perspective of absolute height or length of the skeleton curves, *i.e.*, the effective distance between the horizontal or vertical straight lines in Figure 11a,b, CEW outperformed CIW specimens. The above finding shows that the load-carrying and deformation capacity of CEW were better than CIW. The angle of ultimate displacement for CEW specimens, which is the angle of displacement corresponding to points A_1_ and B_1_, or A_2_ and B_2_ in Figure 11, was larger than CIW. In general, when insulation board is added and externally bonded with single gypsum board, the capacity of elastoplastic deformation for CEW can be significantly improved.

### 4.4. Load-Carrying Capacity and Ductility Coefficient

It is evident from the load-carrying capacity shown in Table 1 that the crack load, yield load and ultimate load for CEW and CIW specimens increased nonlinearly. The values for CEW increased by 1.19 times, 1.02 times and 1.17 times, respectively, compared with CIW specimens. The corresponding displacement and angle of displacement increased nonlinearly as well and the values for CEW increased by 1.14 times, 1.20 times and 1.29 times, respectively, compared with CIW specimens. It is evident from comparison that the load-carrying capacity, displacement and corresponding angle of displacement at key points for CEW specimens outperformed CIW specimens. The above finding suggests that the coordination of insulation board, gypsum boards as well as concrete dense beam and column wall significantly improved the capacity of elastoplastic deformation for CEW specimens. The capacity of deformation for CEW was better than CIW.

**Table 1:** Load-carrying capacity and ductility coefficient.

Parameters		E-1	E-2	E-3	I-1	I-2	I-3
Crack load	*p_cr_* (kN)	78.70	69.43	71.57	60.46	60.11	64.74
	Avg.	73.23			61.77		
Crack displacement	Δ*_cr_* (mm)	0.85	0.75	0.83	0.84	0.69	0.61
	Avg.	0.81			0.71		
Crack displacement angle	*θ_cr_*	1/1741			1/1986		
Yielding force_1	*p_y_* (kN)	209.07	220.25	215.76	212.60	206.40	211.50
	Avg.	215.03			210.17		
Yielding displacement_1	Δ*_y_* (mm)	4.02	4.11	4.16	3.68	3.50	3.07
	*Avg.*	4.10			3.42		
Yield displacement angle	*θ_y_*	1/344			1/412		
Ultimate load	*p_u_* (kN)	369.63	377.71	366.45	318.42	305.02	327.07
	Avg.	371.26			316.84		
Ultimate displacement	Δ*_u_* (mm)	16.08	17.72	18.13	14.61	13.80	11.85
	Avg.	17.31			13.42		
Ultimate displacement angle	*θ_u_*	1/82			1/105		
Ductility ratio_1	*μ* =Δ*_u_/*Δ*_y_*	4.00	4.31	4.36	3.97	3.94	3.86
	Avg.	4.22			3.92		
Elastic stiffness	*α* (kN/mm)	14.03	15.32	19.92	21.81	21.84	22.66
Hardening stiffness	*β* (kN/mm)	2.34	2.55	3.32	3.63	3.64	3.78
Yielding displacement_2	Δ*_y_′* (mm)	3.99	4.53	4.45	4.09	4.02	3.51
	Avg.	4.32			3.87		
Yielding force_2	*p_y_′* (kN)	216.00	210.00	228.00	204.00	209.60	202.80
	Avg.	218.00			205.27		
Ductility ratio_2_Δ*_u_*	*μ′* =Δ*_u_/*Δ*_y_*	4.03	3.91	4.07	3.57	3.43	3.38
	Avg.	4.00			3.46		

Notes: *p_cr_* is the crack load; Δ*_cr_* is the crack displacement; *θ_cr_* is the angle of crack displacement; *p_y_* is the yield load; Δ*_y_* is the yield displacement; *θ_y_* is the angle of yield displacement; *p_u_* is the ultimate load; Δ*_u_* is the ultimate displacement; *θ_u_* is the angle of ultimate displacement.

Ductility coefficient is an important indicator for the seismic performance of composite wallboard [27]. In this study, displacement ductility coefficient (*i.e.*, *μ = Δ_u_/Δ_y_*) described in Specification of Testing Methods for Earthquake Resistant Building (JGJ101-96) was used to analyze the ductility of composite wallboard [18]. It is clear from Table 1 that the average values of ductility coefficients of CEW and CIW were 4.22 and 3.92, respectively. The ductility coefficient of CEW was increased by 7.65% compared with CIW, suggesting that the ductility of CEW was better than CIW and that the capacity of elastoplastic deformation, energy dissipation and seismic performance of CEW was better than CIW.

EN 12512(CEN 2006a) provides another method to compute the yielding point. The elastic branch is drawn through the points on the curve corresponding to 0.1 F_max_ and 0.4 F_max_, whereas the post-elastic branch has a slope equal to 1/6 of that of the first line and is tangent to the curve [19]. The yielding point is obtained by intersection. This method of calculation result is less than the first method, as shown in Table 1. It has a great relationship with the definition of the method. The main advantages of the second method consist of the respecting of the equivalence of strain energy, it is clear that the yielding condition is strongly influenced by the failure condition [19]. 1.14%—the ultimate displacement of new-type of gypsum-concrete composite exterior wallboard; 0.88%—the ultimate displacement of new-type of gypsum-concrete composite interior wallboard. Not only can it provide parameters for an analog overall structure, but it can also facilitate the European specification q-factor calculations.

### 4.5. Damping and Energy Dissipation

Under horizontal cycle load, the seismic performance of CEW and CIW is mainly dependent on the capacity of energy dissipation. The specimens absorb energy when loading and release energy when unloading. The energy dissipation in one cycle is calculated by the difference between the absorption and release of energy. The area of hysteresis loop is therefore used to evaluate the capacity of seismic energy dissipation [28]. If the hysteresis loop is full, the capacity of energy dissipation is high [29].

**Figure 12:**
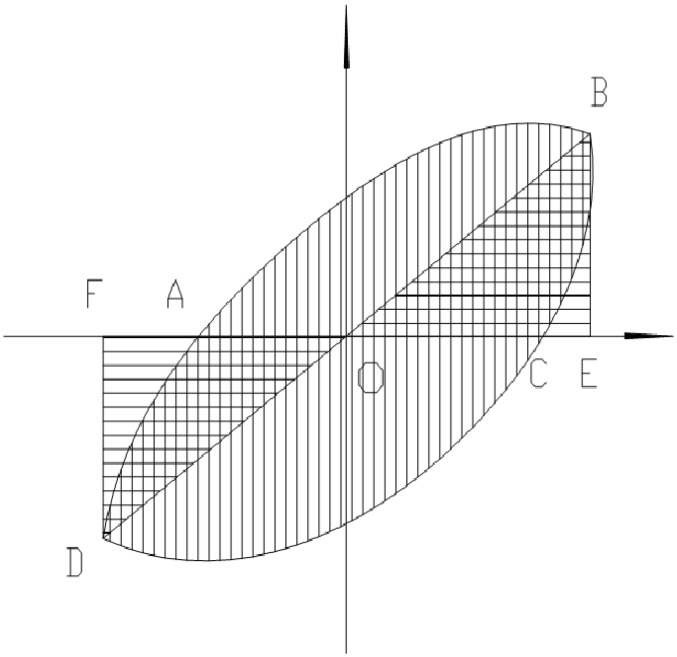
Calculation chart for effective energy and damping ratio.

(1) Representative value of effective energy dissipation E is the total energy enclosed by all surrounded hysteresis loop (*i.e.*, the sum of the energy of all loops : $\sum_{i=1}^{n}S_{ABCD}$) in which *n*, *i* are the number and ordinal of the cycle, respectively; *S_ABCD_* is the dissipated energy in one cycle on the hysteresis curve, as shown in Figure 12. CIW was used as the base of comparison and the relative value of energy dissipation for CIW was defined as 1. According to the representative value of effective energy dissipation shown in Table 2, it was calculated that the relative value of energy dissipation for CEW was 2.68, which was 1.86 times larger than CIW. The above finding suggests that the coordination of insulation board, gypsum boards as well as concrete dense beam and column wall significantly improved the seismic energy dissipation for CEW.

**Table 2:** Viscous damping coefficient and work ratio coefficient.

Specimen	E		*h_e_*								$I_{W}^{S}$							
			1Δ_y_	Avg.	2Δ_y_	Avg.	3Δ_y_	Avg.	1Δ_y_	Avg.	2Δ_y_	Avg.	3Δ_y_	Avg.	
E-1	27,591.11	25,929.34	0.09	0.08	0.08	0.09	0.09	0.09	1.00	1.00	3.65	3.62	8.00	8.32
E-2	25,338.06		0.09		0.09		0.09		1.00		3.63		8.59	
E-3	24,858.84		0.08		0.09		0.09		1.00		3.57		9.63	
I-1	10,318.99	9670.21	0.09	0.07	0.09	0.08	0.10	0.09	1.00	1.00	3.32	3.88	8.37	8.14	
I-2	9706.51		0.07		0.08		0.09		1.00		3.68		7.85		
I-3	8985.12		0.06		0.07		0.07		1.00		4.65		9.63		

Note: 1Δy, 2Δy and 3Δy denotes 1, 2 and 3 times of yield displacement, respectively.

**Figure 13:**
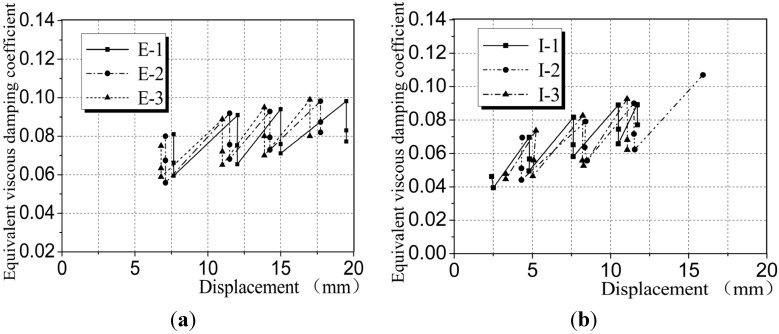
Displacement-equivalent viscous damping coefficient diagram. (**a**) CEW; (**b**) CIW.

**Figure 14:**
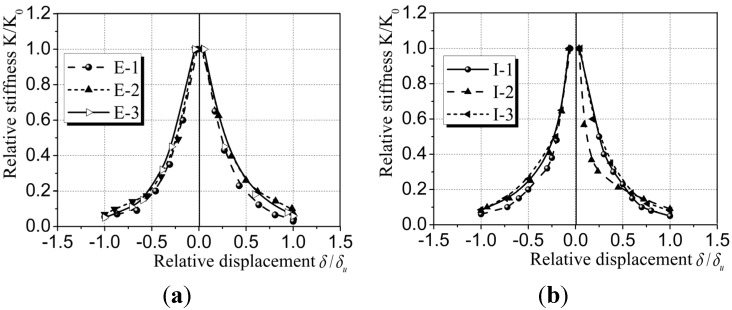
Comparison of stiffness degradation for CEW/CIW. (**a**) CEW; (**b**) CIW.

(2) Equivalent viscous damping coefficient (*h_e_*): equivalent viscous damping coefficient is often used to evaluate the capacity of energy dissipation for wallboard [30]. If *h_e_* is large, the capacity of energy dissipation of the wallboard is high. The calculating equation is *h_e_*= (*S_ABC_ + S_CDA_*)/[(2π *(S_OBE_ + S_ODF_*)], as shown in Figure 12. In which *S_ABCD_* is the dissipated energy in one cycle on the hysteresis curve; *S_OBE_ + S_ODF_* is the area surrounded by hypothetical elastic straight line OB when the same displacement of OD is reached, *i.e.*, the absorbed energy. It is clear from Table 2 that the average values of damping coefficient of CEW and CIW specimens increased steadily from 1Δ to 3Δ_y_, which suggests that the increase of capacity of energy dissipation for both types of wallboard was stable, the coordination between each material was outstanding and the overall load-bearing of the wallboard was reasonable. The increase of *h_e_* indicates the increase of the capacity of elastoplastic deformation. The average value of *h_e_* for CEW was larger than CIW for the first two steps and thus the energy dissipation of CEW outperformed CIW.

(3) Work ratio coefficient $I_{W}^{S}$: Work ratio coefficient is often used to represent the amount of energy absorption and is another indicator of seismic energy dissipation [31]. The equation is: $I_{W}^{S}=\sum_{i=1}^{n}P_{i}\delta_{i}/P_{y}\delta_{y}$, in which *n, i* are the number and ordinal of the cycle, respectively; *p_i_*, *δ_i_* are the load and displacement of the *i*th cycle; *p_y_*, *δ_y_* are the yield load and yield displacement. As shown in Table 2, with the increase of displacement cycle, the average value of $I_{W}^{S}$ increased gradually. For each increment of the displacement cycle, the energy absorbed by the specimens exceeded the energy absorbed during the last cycle. In other words, the capacity of energy absorbance of the wallboard increased and so was the seismic capacity. The value of $I_{W}^{S}$ was increased by 130% and 110% for CEW and CIW, respectively, at 3Δy and 2Δy, suggesting that when entering the elastoplastic stage, the capacity of energy dissipation for CEW in the latter stage outperformed CIW.

(4) Displacement-equivalent viscous damping coefficient diagram: as shown in Figure 13, with the increase of displacement, the equivalent viscous damping coefficients of CEW and CIW were generally stable with a small increase, which suggests that the energy dissipated by the wallboards increased. During the three cycles controlled by displacement, the equivalent viscous damping coefficient reduced, suggesting that the wallboard specimens were damaged during the cyclic loading process and the damage was accumulated. Judging from the overall trends in Figure 13, the curve of CEW was more stable than CIW, suggesting that when CEW was yielded, the stability of load-bearing and deformation was better than CIW.

(5) Stiffness degradation: Stiffness degradation is another indicator of seismic performance for composite wallboard member [32]. It can be used to evaluate the rate of degradation of lateral stiffness of the wallboards, as shown in Figure 14. The slope of line connecting the loading point on the skeleton curve and the origin is defined as the equivalent stiffness *K* of the wallboard. The relative stiffness, *K/K_0_*, is defined as the ratio of equivalent stiffness *K* and initial stiffness *K_0_*. The ratio of displacement *δ* corresponds with the loading point on the skeleton curve to the ultimate displacement is defined as relative displacement, *δ/δu*. It is clear from Figure 14 that during the initial loading stage, due to the fact that new micro-cracks were produced in the wallboard, the stiffness was reduced dramatically. When the angle of top displacement reached 0.27% for CEW and 0.23% for CIW, the wallboards entered a yielding stage. With the increase of load, the amount of newly generated cracks reduced. Existing cracks further extended and widened and the accumulative damage increased. It was suggested that the elastoplasticity of the wallboards was further developed and the lateral stiffness was further degraded. The lateral stiffness at ultimate displacement was reduced to 9% of the initial value. The overall trends of reduction of relative stiffness for CEW was less significant than CIW, suggesting that the capacity of elastoplastic deformation of CEW was better and, thus, the seismic performance of CEW was better than CIW. The initial stiffness of CEW was larger than CIW, indicating that the addition of insulation board and externally bonded single gypsum board contributed to the increase of overall stiffness.

### 4.6. Material Strain

**Figure 15:**
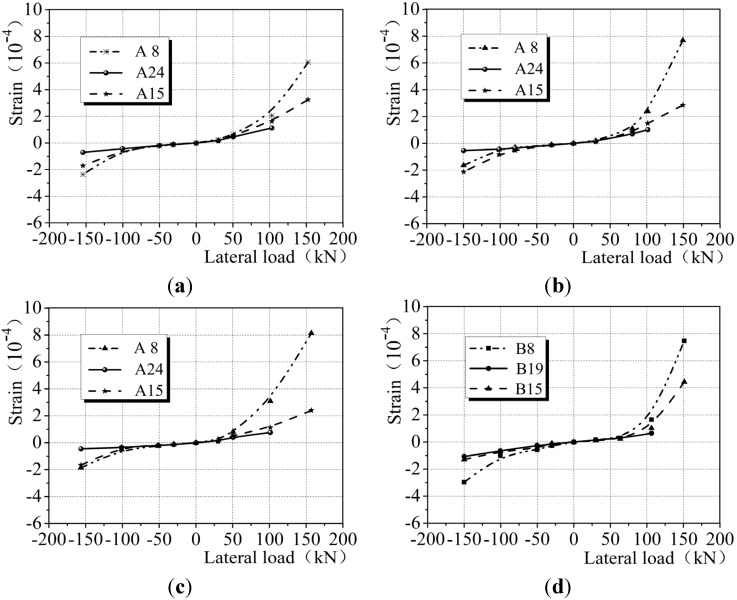
Lateral force-material strain diagram for CEW/CIW. (**a**) E-1; (**b**) E-2; (**c**) E-3; (**d**) I-2.

The analysis of material strain helps evaluate the conditions of coordination of each material as well as the contribution of insulation board and externally bonded single gypsum board towards the concrete dense beam-column wall. Figure 15 shows the lateral force-strain diagram of reinforcement (A8/B8), concrete (A24/B24) and gypsum board (A15/B15) at typical locations for CEW and CIW. A8/B8, A24/B24and A15/B15 located at the same height of the specimens and closed to each other. Stress-strain analysis on the same location can therefore be carried out. It is clear from the experimental results that the concrete of CEW and CIW was cracked under the 4th load step and 1 cycle was conducted for the first 4 load steps. The cracking of concrete led to the failure of strain gauges at the corresponding locations. Therefore, the strain data of key points in Figure 15 sourced from the collected data for CEW and CIW under the first 4 load steps. The load values were sourced from the load on the skeleton curves for the first 4 load steps.

(1) Before the concrete cracked: the strain variation of each material in CEW and CIW was small and can be considered synchronous. At this stage, the reinforcement, concrete, gypsum and insulation of the wallboard were able to work coordinately.

(2) After the concrete cracked: on one hand, on the tension side of the typical location of the wallboard, for CEW and CIW specimens, the strain of the reinforcement increased from 2.52 × 10^−4^ to 7.03 × 10^−4^, and from 2.61 × 10^−4^ to 7.48 × 10^−4^; the strain of gypsum board increased from 1.42 × 10^−4^ to 2.84 × 10^−4^, and from 1.10 × 10^−4^ to 4.97 × 10^−4^. It was suggested that concrete did not carry any load after cracking, which led to the sudden increase of strain in reinforcement and gypsum board. In other words, concrete cracking was the key factor of stiffness and performance degradation of CEW and CIW. The gypsum board and insulation board failed after concrete cracking which suggests that during the initial cracking period, the fiberglass in the gypsum boards effectively resisted tension. The strain of reinforcement for CIW increased by 6.40% compared with CEW and the strain of gypsum increased by 75.00%. It is demonstrated that the insulation board and externally bonded single gypsum board participated in load-carrying which delayed the cracking of concrete dense beam-column wall and prolonged the effective timeframe of CEW. Based on above analysis, the seismic performance of CEW is better than CIW. On the other hand, on the compression side, the variation of strain of each material for CEW and CIW was smaller compared with that of the tension side, which is beneficial for the seismic performance of the wallboard.

**Figure 16:**
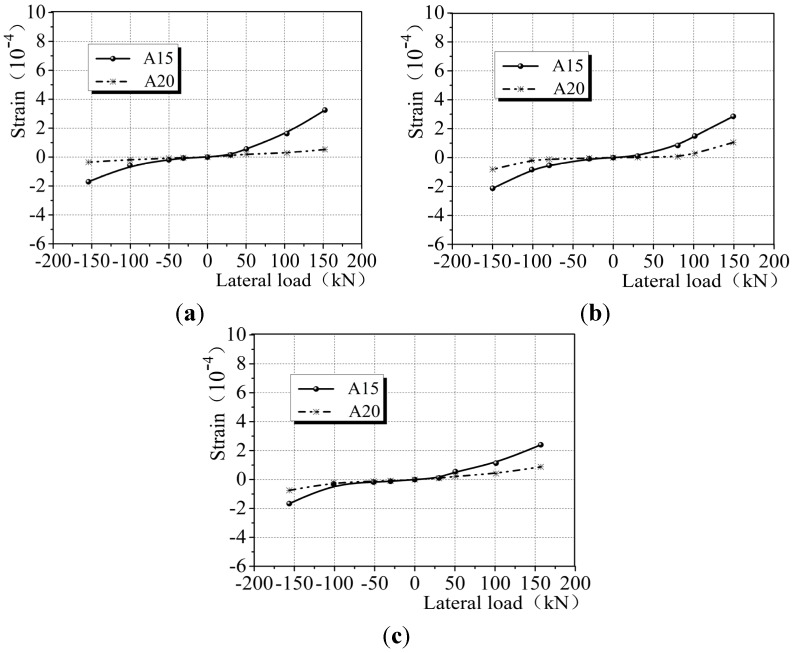
Lateral force-strain diagram in the front and at the back of CEW. (**a**) E-1; (**b**) E-2; (**c**) E-3.

In order to evaluate the load-bearing condition and contribution of CEW gypsum board (front) and externally bonded single board (back), Figure 16 shows the lateral force – strain diagram at A15 and A20 which were located on the typical locations of gypsum boards in the front and back. The range of strain variation at A15 of gypsum board was −1.87 × 10^−4^ to 2.84 × 10^−4^ and at A20 of single gypsum board, −0.64 × 10^−4^ to 0.82 × 10^−4^. It is clear that the load sustained by the gypsum board in the front was 3 to 3.5 times than that at the back. The force sustained by externally bonded gypsum single board was transferred by the insulation board, which suggests that the insulation board sustained a part of force to an extent, and the force sustained exceeded the force carried by externally bonded gypsum board. In general, during the loading process, CEW is able to distribute stress to the insulation board and externally bonded gypsum single board in order to achieve coordination among all materials.

## 5. Conclusions

By means of seismic performance experiment on three new-type composite exterior wallboards and three composite interior wallboards carried out in this study, the following conclusions can be drawn:

(1) A new type of gypsum-concrete composite exterior wallboard was proposed in this study. Horizontal gypsum cavity at every height on the vertical gypsum cavity was setup in the direction of wall height in a concrete dense beam-column lattice structure; concrete was then poured into the gypsum cavity; polystyrene board insulation was setup externally and single gypsum board was bonded to the wallboard. This type of board not only allows for easy setup of vertical and horizontal reinforcement. Compared with conventional concrete-core dense column gypsum board, the load-carrying capacity and lateral force resistance of CEW are significantly improved. Moreover, CEW allows for convenient connection between boards and boards as well as boards and structural columns. Once-off formation is achieved by externally bonded insulation layer and bonded gypsum single board which improves the service life of external insulation.

(2) The analysis of experimental results showed that during the entire loading process of CEW and CIW, the shape of the hysteresis loop transformed from a spindle-shape to “S” shape. Compared with CIW, the ultimate load-carrying capacity of CEW increased by 17.18%, the ductility coefficient increased by 7.65%, the angle of ultimate displacement increased by 28.99% and the effective energy dissipation increased by 1.86 times. The insulation board and externally bonded single gypsum board were found to be beneficial to improve the overall strength and stiffness of CEW. The insulation board, gypsum board and reinforced-concrete dense beam-column worked coordinately which significantly improves the capacity of elastoplastic deformability and seismic energy dissipation for CEW.

(3) The average values of ductility coefficients of CEW and CIW were 4.22 and 3.92, respectively. The values were increased by 124% and 141% for CIW and CEW, when compared with the ductility coefficient *μ_max_* = 1.75 (*i.e.*, have no horizontal gypsum cavity) in reference [9]. It is indicated that CIW and CEW with horizontal gypsum cavity were suitable for seismic members with better ductility. The values were increased by 29.80% and 39.74% for CIW and CEW, when compared with the composite exterior wallboard with the ductility coefficient of 3.02 (*i.e.*, size and vertical reinforcement are exactly the same, and have no horizontal gypsum cavity) in reference [33]. In the paper, *Q* is defined as the increasing ductility coefficient of structural member, *i.e.*, Q = *μ*__eff_/*μ*__code_ in which: *μ*__eff_ is the damage-yield displacement ductility coefficient (*i.e.*, *μ*__eff_ = Δ*u*/Δ*y* of member). *μ*__code_ is defined as the ductility coefficient of specified design structural members in reference [34], *i.e.*, *μ*__code_ = 3.00. As a result, the increasing ductility coefficients of new-type gypsum-concrete composite interior and exterior wallboard are obtained, *i.e.*, *Q_CIW_* = 1.31, *Q_CEW_* = 1.41. It can provide a reference for the seismic and safety design of structural members.

(4) The differential analysis of material strain revealed the contribution of insulation layer and externally bonded single gypsum board to the concrete dense beam-column wall. The gypsum boards in CEW and CIW only participated in load-carrying but due to the existence of fiberglass in the gypsum boards, the cracking of gypsum boards was delayed, and as a result, the cracking of gypsum boards occurred later than concrete. The insulation board and externally bonded single gypsum boards are able to carry a part of the load which delays the cracking and damage of the wallboard and helps improve its deformability and seismic performance.
